# Effective Photodynamic Inactivation of 26 *Escherichia coli* Strains with Different Antibiotic Susceptibility Profiles: A Planktonic and Biofilm Study

**DOI:** 10.3390/antibiotics9030098

**Published:** 2020-02-25

**Authors:** Òscar Gulías, Giselle McKenzie, Miquel Bayó, Montserrat Agut, Santi Nonell

**Affiliations:** 1 Institut Químic de Sarrià, Universitat Ramon Llull, 08017 Barcelona, Spain; giselle.mckenzie@icloud.com; 2 Laboratori d’anàlisis M. Bayó, 08221 Terrassa, Spain; miquel@analisis-bayo.cat

**Keywords:** antimicrobial photodynamic therapy, *Escherichia coli*, uropathogenic strains, planktonic state, biofilm, Methylene Blue

## Abstract

The emergence of multidrug-resistant bacteria is a growing problem and alternative therapies are being sought to effectively address this issue. The aim of this study is to assess a range of *Escherichia coli* strains’ susceptibility to Methylene Blue-mediated antimicrobial photodynamic therapy and determine if this is affected by their antibiotic-resistance profile. Two reference and twenty-four uropathogenic clinical *E. coli* strains were used in this study. All were tested in vitro for antimicrobial susceptibility against sixteen antibiotics. Strains underwent photodynamic treatments using the photosensitizer Methylene Blue with red light and tested in both planktonic and biofilm state. It was found that reference strain ATCC 25922 was susceptible to all tested antibiotics whereas reference strain ATCC 35218 showed resistance only to Ampicillin. With the exception of strains number 16 and 22, all of the isolated strains were multidrug-resistant according to the criteria established by the European Centre for Disease Prevention and Control and the Centre for Disease Control and Prevention, where acquired non-susceptibility to at least one agent in three or more antimicrobial categories is outlined. Photodynamic therapy induced more than 3 log_10_ colony-forming units’ reduction to all strains in planktonic state. Whereas when tested in biofilm state, two and a half times the original dose of methylene blue was necessary to cause a 3 log_10_ antimicrobial effect. There were statistically significant differences in susceptibility among the strains tested in both the planktonic and biofilm experiments. Nevertheless, antimicrobial photodynamic therapy could inactivate all multidrug-resistant strains in the planktonic and biofilm state.

## 1. Introduction

Since the discovery of penicillin by Fleming in 1928, many different families of antibiotics were approved and are currently being used to treat a great variety of infections [1]. However, their therapeutic potential has been threatened by the emergence of increasingly resistant bacterial strains, first observed by Abraham and Chain in 1940. Antimicrobial resistance (AMR) is an increasingly serious threat that endangers human and animal health [2]. It has been estimated that more than 670,000 cases of infections with multidrug-resistant bacteria occurred in the EU in 2015, which caused about 33,000 deaths [3]. Four pathogens have been identified of major concern: cephalosporin-resistant *Escherichia coli*, methicillin-resistant *Staphylococcus aureus* (MRSA), carbapenem-resistant *Pseudomonas aeruginosa* and cephalosporin-resistant *Klebsiella pneumoniae* [3]. There is a gap between the burden of infections due to multidrug-resistant bacteria and the development of new antibiotics to tackle this problem. Therefore, an important research effort is being made to find alternative antimicrobial therapies to which these microorganisms cannot easily develop resistance, such as the use of bacteriophages, antibodies, probiotics, lysins, antimicrobial peptides, genetically modified phages, antibacterial oligonucleotides, and CRISPR-Cas9 [4]. Each of them has advantages and disadvantages relative to current antibiotics and their implementation is currently at different stages. Their main drawbacks are the associated costs, potential toxicity and, in some cases, development of resistance [4].

One additional and actively-explored alternative is antimicrobial photodynamic therapy (aPDT), which is devoid of most of the problems of the above candidates [5]. aPDT uses the combination of light-absorbing chemicals (photosensitizers, PSs), light and oxygen, each devoid of any harmful effects, which result in the production of reactive oxygen species that react with cellular components, consequently leading to microbial cell inactivation. In a clinical setting, aPDT has the benefit of being a safe and effective treatment with the added ability to kill microbial cells rapidly [6], whereas antibiotics can take several days to take effect. Another advantage of aPDT is that the likelihood of developing resistance is considerably low, presumably because of the non-specific, multi-target nature of the damage that leads to cell death [7,8,9,10,11]. Furthermore, some studies have demonstrated that repeated PDT treatments do not induce resistance in the bacteria against aPDT treatment [12,13], although some bacteria may develop tolerance to aPDT upon sub-lethal treatments [14]. The effectiveness of aPDT is limited in situations such as hypoxia or when deeply-seated infections require treatment, the latter is due to the poor penetration of light in tissue. Nevertheless, several strategies are currently being developed to overcome these drawbacks, which do not detract from the intrinsic potential of the photodynamic effect. Moreover, PDT can be used in combination with antibiotics, which aids in enhancing their effect, reducing their dose and hence collateral damages in the surrounding healthy tissues, and even turning susceptive an initially resistant microorganism [15].

A great variety of dyes can efficiently kill Gram-positive bacteria in aPDT, while only cationic PSs, or non-cationic PSs in combination with agents that permeabilize the outer membrane, are able to kill Gram-negative species such as *E. coli* [16]. In this work, we have selected Methylene Blue (MB) as the PS. MB is a clinically-approved cationic dye demonstrated to inactivate several kinds of microorganisms and viruses [17,18].

The susceptibility of antibiotic-resistant strains to aPDT is of interest in the context of AMR because some of the antibiotic resistance mechanisms might affect the effectiveness of aPDT [19,20,21]. Several studies have concluded that aPDT can kill drug-resistant microbes as efficiently as the drug-susceptible ones [22,23,24,25]. However, other studies suggest that the bactericidal effect of aPDT is strain-dependent [26,27]. Thus, the aim of the study is to assess whether the susceptibility of a large variety of antibiotic-resistant strains of *E. coli* to MB aPDT is affected by their antibiotic-resistance profile. The studies are conducted both in planktonic and in biofilm state.

## 2. Results

### 2.1. Antibiotic Susceptibility Profiles

The reference *E. coli* ATCC 25922 was susceptible to all the antibiotics tested, whereas *E. coli* ATCC 35218 showed resistance only to Ampicillin. Table 1 shows the results obtained after performing the antimicrobial susceptibility tests on all of the clinical isolates which details the strains that demonstrated resistance to the tested antibiotics. If a strain is not mentioned in the list, it means that it is susceptible to the drug. The antimicrobial category and the bacterial target of the different tested antibiotics are also specified.

### 2.2. In Vitro Photodynamic Inactivation of E. coli Growing in Planktonic State

**Figure 1:**
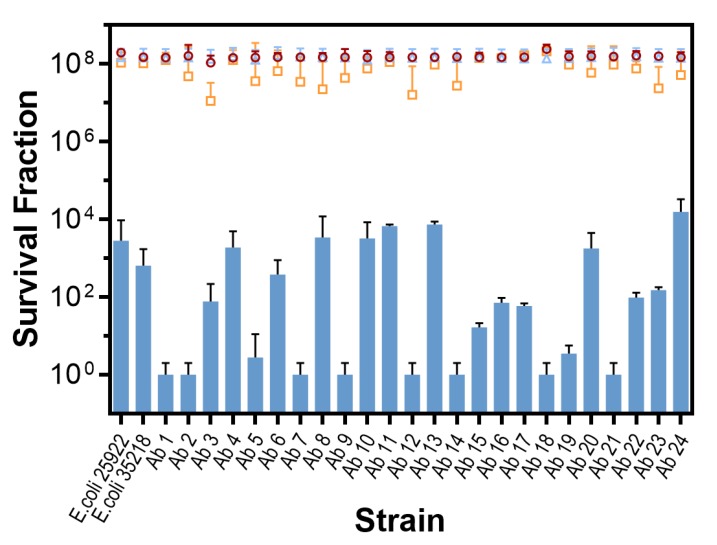
In vitro photodynamic inactivation of *E. coli* growing in planktonic state (18 J/cm^2^ at 625 ± 25 nm, 31 µM MB concentration). Survival fractions are presented as mean + standard deviation (SD). **Circles**: cell control (BS1 -L -MB); **triangles**: light control (BS2 +L -MB); **squares**: MB control (BS3 -L +MB); **bars**: test (BS4 +L +MB).

Using 31 µM of MB and 18 J/cm^2^ of red light, at least a 3 log_10_ CFU/mL (99.9%) reduction was achieved for all strains and more than 6 log_10_ for half of them. Data are shown in Figure 1. Control experiments showed that the effect of light alone is negligible for all strains (*p* > 0.9) while MB alone exerts some toxicity (<1 log_10_; *p* < 0.0001). On the other hand, there were significant differences among the control and the PDT-treated groups (*p* < 0.0001) and there were also significant differences in PDT susceptibility among the strains (*p* < 0.0001).

#### In vitro Photodynamic Inactivation of *E. coli* Growing in Biofilm

**Figure 2:**
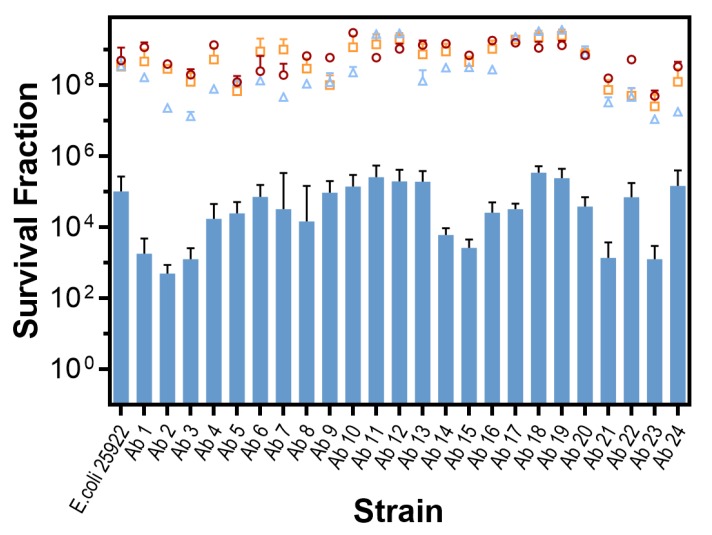
In vitro inactivation of *E. coli* growing in biofilm state (18 J/cm^2^ at 625 ± 25 nm, 78 µM MB concentration). Survival fractions are presented as mean + standard deviation (SD). **Circles**: cell control (BF1 −L −MB); **triangles**: light control (BF2 +L −MB); **squares**: MB control (BF3 −L +MB); **bars**: test (BF4 +L +MB).

All the strains studied in this work grew in biofilm. The photodynamic inactivation treatments using the same conditions employed for the cells in planktonic state were ineffective, therefore the MB concentration was increased. An antimicrobial effect (99.9% or 3 log_10_ CFU/mL reduction) could be achieved for all the strains at a concentration of 78.2 µM. Data are shown in Figure 2.

Control experiments showed that the effect of light or MB alone is negligible for all strains (*p* > 0.9). On the other hand, there were significant differences among the control and the PDT-treated groups (*p* < 0.0001) and there were also significant differences in PDT susceptibility among the strains (*p* = 0.0007).

## 3. Discussion

The susceptibility of 24 clinical isolates of *E. coli* to a panel of antibiotics commonly used in the clinics to treat urinary infections has been studied. The antibiotics were selected from different families with different modes of action. Table 1 shows that the antibiotics nitrofurantoin and tetracycline have the highest number of strains which are resistant (>80%). Moreover, nine out of the sixteen antibiotics tested were ineffective against at least 50% of the clinical strains isolated in this work. In fact, only three antibiotics (cefotaxime, cefoxitin, and ciprofloxacin) were effective in vitro against *all* the isolates. Additional insights can be gained if the data in Table 1 are analyzed in terms of the individual strains (Table 2). All the isolated strains are resistant to at least four antibiotics and one of them (strain 4) is remarkably resistant to eleven out of the sixteen antibiotics. On average, strains were resistant to seven antibiotics. With the exception of strains number 16 and 22, all the isolated strains are multidrug-resistant according to the criteria established by the European Centre for Disease Prevention and Control and the Centre for Disease Control and Prevention, namely acquired non-susceptibility to at least one agent in three or more antimicrobial categories [28]. This does not detract from the fact that strains 16 and 22 show resistance to four of the antibiotics.

In contrast, the results (Figure 1; Figure 2) demonstrate that aPDT exerts an antimicrobial effect (>3 log_10_ CFU/mL reduction) in 100% of the clinical *E. coli* strains tested, irrespective of their antibiotic susceptibility profile, both in planktonic and biofilm state. Detailed inspection of the planktonic state results shows that aPDT achieves a disinfecting effect (>5 log CFU/mL reduction [29]) for 17 isolates, including 15 of the multidrug-resistant strains. It should be noted that these results were obtained with relatively mild conditions, namely 31 µM MB and 18 J/cm^2^ red light, chosen to reveal the different susceptibility of the clinical isolates based on our previous experience with the reference strains and preliminary experiments. Higher MB concentrations or light doses would have resulted in even higher extent of photoinactivation, thereby masking any potential differences among the isolates. Consequently, the study demonstrates that all tested strains of *E. coli*, independent of their antibiotic-resistance profile, can be successfully photo-inactivated by MB, corresponding to previous data for reference strains [30].

Although the bactericidal effect of the treatment is consistent across all strains, there are statistically significant differences in susceptibility amongst them (Figure 1). These results are consistent with those found on *S. aureus* [20]. It has been proposed that some of the resistance factors present in the strains have weight in the susceptibility of *S. aureus* to PDT [31], but no clear correlation has been found between the strains’ response to aPDT and antibiotic resistance.

The situation is less clear in *E. coli*. While some studies had found that the reference strain ATCC 25922 was slightly more susceptible to photoinactivation than multidrug resistant clinical isolates [21,31], our results show that this is the case only for a few isolates (strains 11, 13, and 24), while the majority are significantly more susceptible (Figure 1). Since MB is internalized by *E. coli* [32,33] and is known to be a substrate for efflux pumps in *E. coli* as well as in other bacteria [19,34,35], one could reasonably expect some tolerance to aPDT in the resistant strains. We can speculate that resistance mechanisms such as reduced permeability to antimicrobial agents, active efflux of the antimicrobial from the cell, enzymatic alterations, or degradation of the antimicrobial agent [36] may contribute to modulate the photodynamic activity of MB, but they are not determinants for aPDT efficiency.

Biofilms play a major role in bacterial infections and strongly affect the susceptibility of the bacteria to antibiotics and also to aPDT [37]. *E. coli* biofilms evolve from early stage to mature within 16 h [38], concomitantly increasing the thickness of the extracellular matrix. In our study, biofilms were allowed to grow for at least 16 h to let them reach the mature state. Figure 2 shows the aPDT results obtained for the 24 clinical isolates growing in biofilms. The main finding is that aPDT was able to produce an antimicrobial effect (99.9% kill) on all strains, irrespective of their antibiotic resistance profile. Comparing with the results in planktonic state (Figure 1), it is apparent that photoinactivation is not as efficient and no disinfecting effect (>99.999% kill) could be achieved for any isolate despite increasing the concentration of MB by 2.5-fold. It is well known that photoinactivation of cells living in biofilms require higher concentration of MB compared to their planktonic counterparts [39,40], owing mainly to the mechanical barrier to MB diffusion posed by the extracellular matrix and to alterations in gene expression. As in planktonic cells, statistically significant differences were detected among the strains: Firstly, none of the multidrug-resistant strains appear to be more tolerant to aPDT than the reference ATCC 25922. Thus, as in the planktonic state, the antibiotic-resistance mechanisms do not confer tolerance to aPDT in the biofilm state. In fact, some of the clinical isolates were found more susceptible to aPDT than the reference ATCC 25922 also in biofilm state. Secondly, the differences among strains are less marked than in planktonic state, which indicates that the major determinant of photoinactivation efficacy must be a factor common to all strains, likely the existence of an extracellular matrix. Thus, not surprisingly, no clear correlation is observed between the planktonic and biofilm results.

## 4. Materials and Methods

### 4.1. Bacterial Strains

Twenty-four strains of uropathogenic *E. coli* and two strains obtained from the American Type Culture Collection (*E. coli* ATCC 35218 and *E. coli* ATCC 25922) were tested in this study. The clinical specimens were isolated from twenty-four different anonymized urine samples with pyuria obtained from twenty-four patients on different days after receiving their consent for the use of microorganisms obtained from their samples in investigational work. The patients had received prior instruction in the proper methods for the collection of midstream urine specimens. Specimens were cultured quantitatively for bacteria on brolacin agar (C.L.E.D. agar, Merck, Darmstadt, Germany) and tryptic soy agar (TSA, Merck, Darmstadt, Germany) within one hour of collection unless they were refrigerated. Species identifications were determined after obtaining a pure culture. All the isolated strains were maintained at −80 °C at our premises.

### 4.2. Antibiotic Susceptibility Testing

Antibiotic susceptibility tests of the strains were carried out by means of the disc diffusion method using Rosco Neo-Sensitabs™ tablets commercialized by ROSCO Diagnostica (Taastrup, Denmark). Details of the tested antibiotics and their references are depicted in Table 3. The method was adapted from that described by Kirby-Bauer in 1966 [41]. A few colonies of the strain to be tested were picked from an 18–24 h non-selective agar plate and introduced into a tube containing 4 mL 0.9% NaCl solution obtaining a turbidity equivalent to 0.5 McFarland. One milliliter of this bacterial suspension was then introduced onto a Petri dish containing Mueller-Hinton agar as culture media (Sigma, Saint Louis, United States) and rotated over its entire surface. The surplus suspension was removed using a micropipette. After the inoculum had dried (3–15 min) the antibiotic disks were placed on the agar and the plates were incubated side up in a 35 °C incubator. After 16–18 h incubation, the diameters of the zones of complete inhibition were measured to the nearest whole millimeter to classify the strain as susceptible, intermediate or resistant to each specific antibiotic [42]. Classification of the strains as multidrug-resistant was done according to the criteria established by the European Centre for Disease Prevention and Control (ECDC) and the Centre for Disease Control and Prevention (CDC) [28].

### 4.3. Photosensitizer and Light Source

MB was supplied by Panreac (Montcada i Reixac, Spain). It was dissolved in Milli-Q sterile water to give stock solutions with a dye concentration of 78.2 µM. Stock solution was stored in the dark for no more than 1 week and was diluted in PBS immediately before experiments. Red light at 625 ± 25 nm was delivered using an 18 LED light source (Showtec, Par64 Short, Sussex, UK).

### 4.4. Photodynamic Inactivation of E. coli Growing in Planktonic State:

For each strain, three independent tests were carried out as follows: Bacteria were grown aerobically overnight at 37 °C in Tryptic Soy Broth (TSB, Panreac 413820.1210, Castellar del Vallès, Spain) in an orbital shaking incubator. A volume of 10 µL of this pre-inoculum was added to 10 mL of sterile TSB and incubated, until the culture reached an optical density of 0.5 at 600 nm (OD600, measured in a Jenway 6305 spectrophotometer), corresponding to ca. 1.5 × 10^8^ CFU/mL.

The suspensions were then centrifuged (5 min, 3000 rpm) and the pellet was resuspended in 5 mL of phosphate-buffered saline at pH 4 (PBS, Fisher BP399, New Jersey, USA). Two 0.5 mL aliquots of this bacterial suspension were then diluted with PBS to 1 mL (BS1 and BS2) and two additional 0.5 mL aliquots were diluted with a 62 µM MB solution in PBS also to 1 mL (BS3 and BS4; final MB concentration 31 µM). The four resulting suspensions were then incubated in the dark in an orbital shaking incubator (Rotabit-JP Selecta) at 37 °C for 15 min and 60 rpm.

Then, three 300 µL aliquots of BS1 and BS3 were placed in different wells of a 96-well plate and kept in the dark until the end of the experiments (dark controls BS1 -L -MB and BS3 -L +MB, respectively). In turn, three 300 µL aliquots of BS2 and BS4 were placed in different wells of a second 96-well plate and irradiated with red light (625 ± 25 nm, 8 mW/cm^2^, 18 J/cm^2^) 25 cm from the top of the plates (light control BS2 +L -MB and test BS4 +L +MB, respectively).

At the end of the experiments, all suspensions were ten-fold serially diluted in PBS in triplicate, and 10 µL of each dilution was streaked on Tryptic Soy Agar plates (TSA, TSB added with 1.5% agar-agar, Fisher BP9744, Geel, Belgium) and incubated aerobically in the dark for 18 to 20 h at 37 °C. After the incubation period, the number of CFUs/mL was determined, yielding 9 results for each of the four BSs. These conditions were selected based on our previous experience with the reference *E. coli* strains.

### 4.5. Photodynamic Inactivation of E. coli Growing in Biofilm

This assay was performed against the reference *E. coli* ATCC 25922, a strain known to produce biofilms [43], along with the clinical strains of uropathogenic *E. coli*. For all strains, it was checked that they were capable to live in biofilm as described by Crémet et al. [44].

For each strain, three independent tests were carried out as follows: Bacteria were grown aerobically overnight (minimum 16 h) at 37 °C in TSB in an orbital shaking incubator. Twelve 2-µL aliquots of this pre-inoculum were placed in the wells of two different 96-well polystyrene plates (six aliquots in each plate) containing each 130 µL of TSB and were incubated overnight at 37 °C without stirring to allow the formation of biofilms (BFs).

The supernatant liquid was then removed from the wells, and the benthonic cells were gently washed three times with sterile PBS solution. After the third wash, three wells in each plate were refilled with 130 µL of PBS (BF1 and BF2, respectively), while the other three were refilled with a 78 µM MB solution (BF3 and BF4, respectively).

The BFs were then incubated at 37 °C for 15 min in darkness. After the incubation period, the plate containing the three replicates of BF1 and BF3 was kept in the dark until the end of the experiments (dark controls BF1 -L -MB and BF3 -L +MB, respectively), while the plate containing the three replicates of BF2 and BF4 was irradiated with red light (625 ± 25 nm, 8 mW/cm^2^, 18 J/cm^2^) 25 cm from the top of the plates (light control BF2 +L -MB and test BF4 +L +MB, respectively).

At the end of the experiments, all BFs were washed three times with PBS and the remaining surface-attached cells of the wells were resuspended with 180 µL of PBS by pipetting up and down. All suspensions were then ten-fold serially diluted in PBS in triplicate, and 10 µL of each dilution was streaked on Tryptic Soy Agar plates and incubated aerobically in the dark for 18 to 20 h at 37 °C. After the incubation period, the number of CFUs/mL was determined, yielding 9 results for each of the four BFs.

### 4.6. Statistics

Survival fractions are presented as mean + standard deviation (SD). Differences between the means were compared for significance by a one-way ANOVA using the GraphPad Prism 7.04 software. Values of *p* < 0.05 were considered significant.

## 5. Conclusions

The usefulness of photodynamic therapy against 26 clinical isolates of *E. coli* with different antibiotic susceptibility profiles, most of them multidrug resistant, has been demonstrated. aPDT was able to exert an antimicrobial effect in 100% of the strains, both in planktonic and biofilm state, with significant differences in susceptibility among strains. Antibiotic resistance did not confer tolerance to aPDT in any of the clinical isolates tested.

## Figures and Tables

**Table 1:** Resistance profile of the tested clinical strains of uropathogenic *Escherichia coli* to various antibiotics.

Antimicrobial Category	Antibiotic	Bacterial Target	Identification Number of the Resistant Strains	% of Resistant Strains
Penicillins	Ampicillin	Cell wall synthesis	1–7, 9–14, 17, 19, and 20	67
Penicillins + β-lactamase inhibitors	Amoxicillin + Clavulanic acid		21 and 23	8
First-generation Cephalosporins	Cephalotin		1, 2, 4, 6–10, 14–24	79
Second-generation Cephalosporins	Cefuroxime		1, 6–8, 14, 17, 19, and 20	33
Third-generation Cephalosporins	Cefotaxime		None of them	0
Cephamycins	Aztreonam		1, 4–9, 12, 14–16, 18, 22, and 24	58
	Cefoxitin		None of them	0
Quinolones	Ciprofloxacin	DNA gyrase	None of them	0
	Nalidixic acid		3, 4–9, 11, 13, 15–20, 22, and 24.	71
	Norfloxacin		4	4
Macrolides	Azithromycin	50S subunit of the ribosome	4, 9, 17, 19, and 20	21
Tetracyclines	Tetracycline	30S subunit of the ribosome	1–15, 18, 20, 21, 23, and 24.	83
Aminoglycosides	Gentamycin		1–4, 6, 7, 9, 10, 13, 14, 17, and 19	50
Phosphoenolpyruvates	Fosfomycin	UDP-N-acetylglucosamine enolpyruvyl transferase	1, 2, 4, 5, 8–12, 14, 15, 17–20, and 24	67
Furantoins	Nitrofurantoin	Various bacterial enzymes and DNA	2–13, 15–19, 21–24	88
Diaminopyrimidine + Sulfamide	Trimethoprim + Sulfamethoxazole	Synthesis of folic acid	1–5, 8, 11–15, 18, 20, 21, 23, and 24.	67

**Table 2:** Resistance profile of the tested clinical strains of uropathogenic *Escherichia coli* to various antibiotics.

Antibiotic	Strains																							
	1	2	3	4	5	6	7	8	9	10	11	12	13	14	15	16	17	18	19	20	21	22	23	24
Ampicillin	R	R	R	R	R	R	R		R	R	R	R	R	R			R		R	R				
Amoxicillin + Clavulanic acid																					R		R	
Cephalotin	R	R		R		R	R	R	R	R				R	R	R	R	R	R	R	R	R	R	R
Cefuroxime	R					R	R	R						R			R		R	R				
Cefotaxime																								
Aztreonam	R			R	R	R	R	R	R			R		R	R	R		R				R		R
Cefoxitin																								
Ciprofloxacin																								
Nalidixic acid			R	R	R	R	R	R	R		R		R		R	R	R	R	R	R		R		R
Norfloxacin				R																				
Azithromycin				R					R								R		R	R				
Tetracycline	R	R	R	R	R	R	R	R	R	R	R	R	R	R	R			R		R	R		R	R
Gentamycin	R	R	R	R		R	R		R	R			R	R			R		R					
Fosfomycin	R	R		R	R			R	R	R	R	R		R	R		R	R	R	R				R
Nitrofurantoin		R	R	R	R	R	R	R	R	R	R	R	R		R	R	R	R	R		R	R	R	R
Trimethoprim + Sulfamethoxazole	R	R	R	R	R			R			R	R	R	R	R			R		R	R		R	R
Number of resistances	8	7	6	11	7	8	8	8	9	6	6	6	6	8	7	4	8	7	8	8	5	4	5	7
Multidrug resistant?	Y	Y	Y	Y	Y	Y	Y	Y	Y	Y	Y	Y	Y	Y	Y	N	Y	Y	Y	Y	Y	N	Y	Y

**Table 3:** Antibiotics tested against the *E. coli* strains.

Antibiotic	Dose/µg	Reference of the Rosco Neo-Sensitabs™ tablets
Ampicillin	10	567NR 60212
Amoxicillin + Clavulanic acid	20 + 10	567NR 60112
Cephalothin	30	567NR 60612
Cefuroxime	30	567NR 60512
Cefotaxime	30	567NR 63912
Aztreonam	30	567NR 63612
Cefoxitin	10	567NR 62912
Ciprofloxacin	5	567NR 60812
Nalidixic acid	30	567NR 61412
Norfloxacin	10	567NR 76212N
Azithromycin	15	567NR 60312
Tetracycline	30	567NR 62012
Gentamycin	10	567NR 61112
Fosfomycin	200	567NR 62312
Nitrofurantoin	300	567NR 62612
Trimethoprim + Sulfamethoxazole	1.25 + 23.7	567NR 62212
